# Protective and curative effects of unconjugated bilirubin on gene expression of LOX-1 and iNOS in the heart of rats receiving high-fat diet and low dose streptozotocin: a histomorphometric approach

**DOI:** 10.1186/s12950-024-00397-8

**Published:** 2024-07-09

**Authors:** Mohammad Hasan Maleki, Omid Vakili, Ramin Tavakoli, Elham Nadimi, Zahra Noori, Motahareh Taghizadeh, Amirreza Dehghanian, Lobat Tayebi, Sayed Mohammad Shafiee

**Affiliations:** 1 Department of Clinical Biochemistry, School of Medicine, Shiraz University of Medical Sciences, Shiraz, Iran; 2 Autophagy Research Center, Department of Clinical Biochemistry, School of Medicine, Shiraz University of Medical Sciences, Shiraz, Iran; 3 Department of Clinical Biochemistry, School of Pharmacy and Pharmaceutical Sciences, Isfahan University of Medical Sciences, Isfahan, Iran; 4 Student Research Committee, School of Medicine, Shiraz University of Medical Sciences, Shiraz, Iran; 5 Histomorphometry and Stereology Research Center, Shiraz University of Medical Sciences, Shiraz, Iran; 6 Department of Anatomical Sciences, School of Medicine, Shiraz University of Medical Sciences, Shiraz, Iran; 7 Trauma Research Center, Shiraz University of Medical Sciences, Shiraz, Iran; 8 Molecular Pathology and Cytogenetics Division, Department of Pathology, School of Medicine, Shiraz University of Medical Sciences, Shiraz, Iran; 9 Marquette University School of Dentistry, Milwaukee, WI 53233 USA

**Keywords:** Atherosclerosis, Bilirubin, High fat diet, Cell adhesion molecules, Heart, Oxidative stress

## Abstract

**Background:**

Atherosclerosis is a chronic inflammatory condition affecting the large arteries and is a major cause of cardiovascular diseases (CVDs) globally. Increased levels of adhesion molecules in cardiac tissue serve as prognostic markers for coronary artery occlusion risk. Given the antioxidant properties of bilirubin and its inverse correlation with atherosclerosis, this study aimed to assess the beneficial effects of bilirubin on atherosclerotic indices and heart structure in high-fat diet-fed diabetic rats with atherosclerosis.

**Methods:**

Atherosclerosis was induced in three out of five groups of adult male Sprague Dawley rats through a 14-week period of high-fat diet (HFD) consumption and a single low dose of streptozotocin (STZ) (35 mg/kg). The atherosclerotic rats were then treated with intraperitoneal administration of 10 mg/kg/day bilirubin for either 6 or 14 weeks (treated and protected groups, respectively), or the vehicle. Two additional groups served as the control and bilirubin-treated rats. Subsequently, the mRNA expression levels of vascular cell adhesion molecule 1 (VCAM-1), intercellular adhesion molecule 1 (ICAM-1), lectin-like LDL receptor 1 (LOX-1), and the inducible nitric oxide synthase (iNOS) were analyzed using quantitative reverse transcriptase-polymerase chain reaction (qRT-PCR). Histopathological and stereological analyses were performed to assess changes in the heart structure.

**Results:**

Bilirubin significantly decreased the expression of VCAM-1, ICAM-1, LOX-1, and iNOS genes in the treated group. Moreover, bilirubin mitigated pathological damage in the left ventricle of the heart. Stereological analysis revealed a decrease in the left ventricle and myocardium volume, accompanied by an increase in vessel volume in rats treated with bilirubin.

**Conclusion:**

These findings demonstrate that mild hyperbilirubinemia can protect against the progression of atherosclerosis and heart failure by improving lipid profile, modulating adhesion molecules, LOX-1, and iNOS gene expression levels.

## Introduction

Atherosclerosis is a progressive and chronic inflammatory disorder characterized by various pathological processes, including endothelial dysfunction, formation of foam cells, and accumulation of lipid deposits. These processes ultimately contribute to the development of intravascular lesions [1]. . While hyperglycemia is recognized as a major contributor to the progression of atherosclerosis in diabetic patients, it is important to acknowledge that additional risk factors, such as dyslipidemia and hypertension, can exacerbate vascular damage through synergistic effects [2]. .

According to the previously conducted evaluations, there is an interesting relationship between serum bilirubin levels and coronary artery disease (CAD) development. In this context, individuals with higher serum bilirubin content have demonstrated a lower risk of CAD and CVD progression. Moreover, serum bilirubin concentrations are inversely correlated with the incidence of inflammatory and oxidative stress-induced diseases, such as atherosclerosis [3–5]. Bilirubin in brief, is synthesized during the conventional catabolism of heme-by-heme oxygenase (HO). Bloodstream bilirubin exists in two primary forms, including unconjugated (indirect) and conjugated (direct) bilirubin. Unconjugated bilirubin, a byproduct of red blood cell breakdown, travels to the liver for further processing. However, its water-insoluble nature necessitates binding to albumin for transport. Upon reaching the liver, unconjugated bilirubin undergoes a chemical transformation, called glucuronidation mediated by UGT1A1 UDP-glucuronosyltransferase, becoming water-soluble conjugated bilirubin. This conjugated form is then eliminated through the stool. A bilirubin test measures both unconjugated and conjugated bilirubin, along with total bilirubin, to assess liver function and its ability to process and eliminate bilirubin [6]. Once the HO isoform, i.e. HO-1, is induced, it attenuates the inflammation and inflammatory processes contributing to atherogenesis [7–9]. It seems that HO-1 activation is an adaptive response against the ROS production, due to the production of unconjugated bilirubin that is a potential endogenous antioxidant [7].

VCAM-1 plays a pivotal role in the development of atherosclerosis. The migration of leukocytes into the vascular intima, which is a crucial early event in plaque formation, is facilitated by the presence of VCAM-1 on the surface of activated endothelial cell [10]. ICAM-1 is another up-regulated endothelial adhesion molecule at the site of atherosclerosis; studies have indicated that the levels of soluble ICAM-1 are correlated with the extent of atherosclerosis [11, 12]. It has been reported that ICAM-1 knockdown is in association with a decrease in the size of vascular lesions in apolipoprotein E-deficient mice [13]. The overproduction of oxLDLs also seems to be essential in the development of atherosclerosis and endothelial dysfunction through several mechanisms, such as the overexpression of cell adhesion molecules (CAMs), the imbalanced activation of endothelial cells nitric oxide synthase (eNOS), and the hyper-activation of the inducible nitric oxide synthase (iNOS), which in turn enhances the inflammatory processes within the vascular intima [14–16]. Desirably, HO preserves vascular nitric oxide (NO) levels and bilirubin is directly related to vascular NO content [17].

In the case of LOX-1, it is a scavenger receptor that selectively internalizes the oxLDLs into endothelial cells to mediate vascular inflammation. As a type II transmembrane protein, LOX-1 has a high affinity for oxLDLs and its subsequent interaction with oxLDLs would significantly stimulate the vascular expression of LOX-1 [18–20]. High levels of oxLDLs result in the interruption of the eNOS/iNOS balance, the over expression of LOX-1, and consequent inflammation of vascular tissues, leading to the enhanced endothelial adhesiveness of leukocytes and the development of atherosclerosis. LOX-1/oxLDLs interaction also contributes to an inflammatory response induced by the overexpression and activation of adhesion molecules, including VCAM-1 and ICAM-1, chemotactic factors (e.g., MCP-1), and ROS formation [21].

In this study, our objective was to investigate the effects of bilirubin administration on atherosclerosis, both during its development and after its establishment, using rat models induced by a HFD and low doses of STZ. Our aim was to explore the potential of this endogenous antioxidant for both prevention and treatment of atherosclerosis. We analyzed the expression levels of key atherosclerosis-related genes, namely ICAM-1, VCAM-1, iNOS, and LOX-1, to assess the molecular impact of bilirubin. Additionally, we measured serum biochemical indices, including liver enzymes, lipid profile, and fasting blood glucose (FBG). Furthermore, histopathological and stereological studies were conducted to evaluate any potential changes in the structure of the heart in the atherosclerosis models.

## Materials and methods

### Animals and experimental design

An HFD, containing 83% fat, 11% carbohydrate, and 6% protein with a total caloric value of 5420Kcal, was prepared to feed the animals (Table 1) [22–24]. To overcome bilirubin insolubility (Sigma-Aldrich, USA), the modified oleic acid was employed [25, 26]. In this context, 10 mg/kg/day (17 µmol/kg/day) of injectable bilirubin solution (dissolved in modified oleic acid) was determined as the suitable dose for intraperitoneal (IP) injection. An almost 2-fold increase was observed in serum levels of total bilirubin following the injection of the aforementioned dose. In a pilot study, we also examined the effects of injecting 20 and 30 mg/kg/day bilirubin doses to each rat, and after one week, the animal mortality increased. Thus, the appropriate concentration for our study was opted based on the viability of rats during the pilot study.

**Table 1:** The composition and caloric content of the emulsified HFD

Components of high-fat emulsion	Amount	Energy
Fat		
Corn oil	400 (g)	3600 Kcal
Cholesterol	100 (g)	900 Kcal
**Carbohydrate**		
Saccharose	150 (g)	600 Kcal
**Protein**		
Total milk powder	80 (g)	320 Kcal
**Other ingredients**		
Sodium deoxycholate	10 (g)	-
Tween 80	36.4 (g)	-
Propylene glycol	31.1 (g)	-
Vitamin mixture	2.5 (g)	-
Cooking salt	10 (g)	-
Mineral mixture	1.5 (g)	-
Distilled water	300 (ml)	-
**Total energy**	**-**	**5420 (Kcal)**

**Fig. 1:**
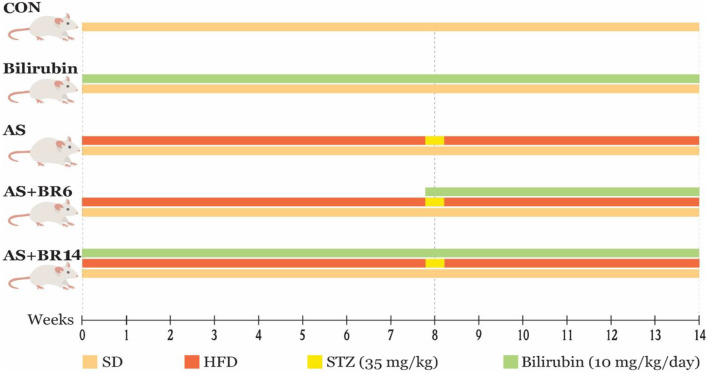
A schematic view of rats treatment during the experiment. The animals were divided into five groups (*n* = 6/ group), including the Control group (CON), receiving standard laboratory diet and oleic acid as the vehicle (0.3 ml per rat); the Bilirubin group (B), receiving standard laboratory diet with an intraperitoneal injection (IP) of 10 mg/kg/day of bilirubin (previously dissolved in oleic acid); the Atherosclerosis group (AS), receiving high-fat diet (HFD), with IP of 35 mg/kg of STZ (freshly prepared by dissolving in citrate buffer 0.1 mol/L; pH 4.5 at the 8th week; and the Treated group, (AS + BR6), receiving HFD for 14 weeks and IP injection of 35 mg/kg of STZ at the 8th week with IP injection of 10 mg/kg/day of bilirubin at the 8th week which continued to the 14th weeks (6 weeks); and Protected group (AS + BR14), receiving HFD for 14 weeks and IP injection of 35 mg/kg of STZ at the 8th week with IP injection of 10 mg/kg/day of bilirubin from the beginning of the experiment (14 weeks)

Thirty adult male Sprague Dawley rats, weighing 150–200 g, were purchased from the Animal Breeding Center of Shiraz University of Medical Sciences. During familiarization time (one week), the animals were kept under the standard environmental conditions (12:12 h dark/light cycle, temperature at 25 ± 2 °C), and they had open access to standard laboratory food and water. All experiments conducted in animal laboratory were confirmed by the University Ethics Committee and performed according to the regulations for using and caring for experimental animals at Shiraz University of Medical Sciences (**IR.SUMS.AEC.1402.030**). The animals were divided into five groups (*n* = 6/ group), including the Control group (**CON**), receiving standard laboratory diet and oleic acid as the vehicle (0.3 ml per rat); the Bilirubin group (**B**), receiving standard laboratory diet with an intraperitoneal injection (IP) of 10 mg/kg/day of bilirubin (previously dissolved in oleic acid) [25, 26]; the Atherosclerosis group (**AS**), receiving high-fat diet (HFD) through gavage (Table 1), with IP of 35 mg/kg of STZ (freshly prepared by dissolving in citrate buffer 0.1 mol/L; pH 4.5 at the 8th week [22–24]; the Treated group, (**AS + BR6)**, receiving HFD for 14 weeks and IP injection of 35 mg/kg of STZ at the 8th week with IP injection of 10 mg/kg/day of bilirubin at the 8th week which continued to the 14th weeks (6 weeks); and the Protected group **(AS + BR14)**, receiving HFD for 14 weeks and IP injection of 35 mg/kg of STZ at the 8th week with IP injection of 10 mg/kg/day of bilirubin from the beginning of the experiment (14 weeks) (Fig. 1).

Ten milligrams of bilirubin per kilogram of rats’ weight (on average, for every four recipient rats at the beginning of the experiment) were dissolved in the minimum amount of oleic acid as vehicle, until no bilirubin residue remained, and was injected intraperitoneally (IP) among the rats daily at an average volume of 0.3 ml per rat based on their weight.

The selection of the 10 mg/kg/day dosage of bilirubin (equivalent to 17 µM/kg/day) was specifically informed by the serum bilirubin levels observed in patients with Gilbert’s syndrome and previous assessments also confirmed the suitability of this dosage [27, 28].

During the 14 weeks experiment, the animals were weekly weighted.

At the end (14 weeks) of the experiment, the animals were anesthetized with 100 mg/kg ketamine and 10 mg/kg xylazine (Merck, Germany), and then they were euthanized in a CO_2_ box (based on the ethical guidelines). Blood samples were collected from the animals’ heart for further biochemical analyses. The heart tissue was removed, and the left ventricle was fixed in 10% formalin buffer for histopathological and stereological study. The remaining tissues of the heart and aorta were kept in the liquid nitrogen for q RT-PCR analysis.

### Measurement of biochemical parameters

Blood collected in tubes was centrifuged at 3000 rpm for 15 min. The collected serum was stored at − 20 °C for the determination of some biochemical serum parameters.

Subsequently, serum levels of FBG and lipid profile, including triglycerides (TG), total cholesterol (Chol), high density lipoprotein (HDL), and low density lipoprotein (LDL), as well as liver markers, including aspartate aminotransferase [AST], alanine aminotransferase (ALT), alkaline phosphatase (ALP), total protein (TP), total bilirubin (TBIL), direct bilirubin (DBIL), and albumin (Alb) were measured using the Pars Azmoon diagnostic kit (Tehran, Iran), according to the manufacturer’s instructions by the Perestig24i auto-analyzer.

### Quantitative reverse transcriptase- polymerase chain reaction assessments

The mRNA expression levels of VCAM-1, ICAM-1, iNOS, and LOX-1 were analyzed by the q RT-PCR method using the ABI 7500 real-time PCR system. For this purpose, total RNA was extracted from the aorta using the TRIzol extraction kit (Yekta Tajhiz Azma, Tehran, Iran). The extracted RNA was then reverse transcribed into cDNA using a cDNA synthesis kit (Yekta Tajhiz Azma, Tehran, Iran). The β-actin expression levels were separately measured in each sample to be considered as the endogenous control. Amplification reactions were conducted by the SYBR Green master mix (Amplicon, Denmark). All samples were run in triplicate and the mean value of triplicates was used in analyses. Deionized distilled water (ddH2O) was served as a no-template control (NTC). Finally, the relative quantity of the corresponding mRNA was analyzed by the comparative Ct (2^−ΔΔCt^) method. The Allele ID software (version 7.73) was applied to design the primers’ sequences (Table 2).

**Table 2:** List of primers’ sequences used for q RT-PCR analyses

Gene	Size (bp)	Forward primer	Reverse primer
*VCAM-1*	114	5΄-AAGTGGAGGTCTACTCATTCC-3΄	5΄-GGTCAAAGGGGTACACATTAG-3΄
*ICAM-1*	152	5΄-CGACTGGACGAGAGGGATTG-3΄	5΄-GGAGAGCACATTCACGGTC-3΄
*iNOS*	121	5΄-TTCTTTGCTTCTGTGCTAATGCG-3΄	5΄-GTTGTTGCTGAACTTCCAATCGT-3΄
*LOX-1*	144	5΄-ATTGTACAGCAGACACAGTTACTC-3΄	5΄-GTTCCCTCTTTGATTCTTGTGAAG-3΄
*β-actin*	207	5΄-ATTGCTGACAGGATGCAGAA-3΄	5΄-TAGAGCCACCAATCCACACAG-3΄

### Stereological and histopathological studies

The unbiased stereological evaluations were used to estimate the structural changes related to the heart [29]. The volume of the left ventricles (LV) was estimated and then divided into 8 to 12 isotropic uniform random (IUR) sections using the orientation method [30–32]. Tissues were embedded and the sections at 5 and 25 μm thickness were prepared, and then stained with Hematoxylin and Eosin (H&E) [33]and Trichrome Masson [34]. The resulting slides were used for quantitative stereological analysis, including estimating shrinkage, left ventricular volume, and total volume of the myocardium, endocardium, and vessels. A pathologist examined the sections to identify pathological indices such as the thickness of the myocardial layer, the hypertrophy of cardiomyocyte bundles, the connective tissue between cardiac muscle bundles, and vascularity. The observer examined at least ten fields in each slide.

### Estimating the left ventricular volume

The heart was sectioned, and the volume of its left ventricle was estimated using the Weibel method [35]. Briefly, a bottle filled with isotonic saline was placed on a balance and weighed, and the tissue was then kept hold on a fine thread inside the container. The left ventricular volume was estimated using the following formula:

Volume _(left ventricle)_ = (W2-W1)/SG.

Where “W2” and “W1” represent respectively into the left ventricle, the weight of the saline-filled section before and after immersing, and “SG” is the specific gravity of the isotonic saline (1.0048 g/cm^3^). It should be noted that weight in “g” was equal to volume in “cm^3”^.

### Estimating the shrinkage and the total volume of the myocardium, endocardium, and the vessels, along with histopathological evaluations

The organ shrinkage was calculated using a trocar by preparing single slices from the Sect. [25]. The mean values of their radius were calculated as the pre-fixing radius (R_before_). After tissue processing and staining, the two vertical diameters in the above section-parts were re-measured, and the mean values of their radius were calculated as the post-fixing radius (R_after_). The shrinkage volumes of the tissues were then calculated using the following formula:

Volume _shrinkage_ = 1 – (R_after_/R_before_)^1.5^.

The orientation method was used to obtain Isotropic Uniform Random (IUR) sections. The IUR slices of the left ventricle were obtained by placing the tissue on an equally divided circle plate (ɸ) and cutting it into two parts in random directions. Left ventricular pieces were sliced into new random directions after being placed on a cosine-weighted splitting circle plate (ɵ).

**Fig. 2:**
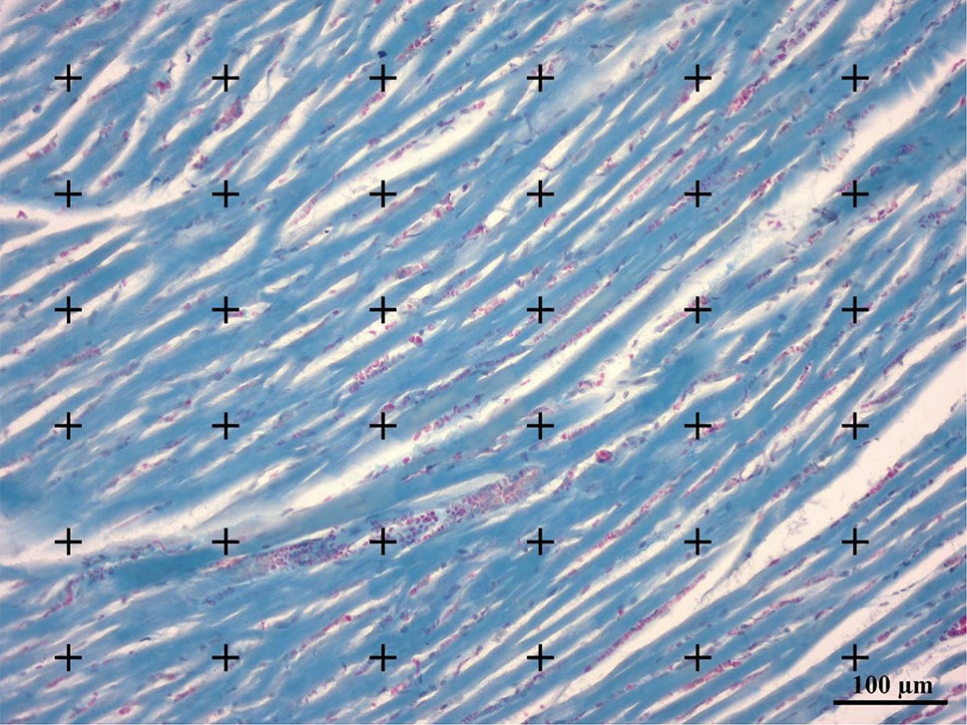
Point-counting method for estimating the volumetric density of the myocardium, endocardium, and blood vessels by dividing the number of the points hitting the structure by the total number of the points

For histopathological investigations, the slices and a circle punched out together were embedded in blocks and sectioned with a microtome. The paraffinized left ventricles were cut into 5-µm thick sections and co-stained with hematoxylin and eosin (H & E) (Merck, Germany). Afterwards, the areas were analyzed using a video-microscopy system that consisted of a microscope (E-200, Nikon, Japan) linked to a video camera (SSC, Sony, Japan), a computer, and a flat-screen monitor. The point-counting method was used to estimate the volumetric density of the preferred structures containing myocardium, endocardium, and the vessels (Fig. 2).

To estimate the “Vv _(Structures/left ventricle)_” volume density of myocardium, endocardium, and the vessels, the point counting method on 5-µm sections was applied. The total number of the points that randomly hit the myocardium, endocardium, and the vessels was calculated and then divided by the total number of the points that hit the whole left ventricle. The total volume of each structure was obtained using the below formula:

Vv_(Structure)_ = ∑P_(Structure)_/ ∑P_(Left ventricle)_.

Where ΣP_(structure)_ was the number of grid intersection points with each of the myocardium, endocardium, and the vessels tissues, and ΣP_(left ventricle)_ was the number of grid intersection points with the left ventricle of the heart tissue.

The final volume was then calculated using the following formula:

Absolute V_(structure)_ = Vv_(Structure)_ × V_(left ventricle)_.

### Statistical analysis

The values were expressed as mean ± SD. Quantitative results were analyzed by the Kruskal-Wallis test followed by Dunn’s post hoc test for multiple comparisons using the SPSS (version 24.0) and the GraphPad Prism Software (version 9.0.0; La Jolla, CA, USA). The two-way repeated measure ANOVA test was used to evaluate the rats’ body weight changes during the study. When the values of p were lower than 0.05, the difference was considered statistically significant.

## Results

### Effects of HFD and Bilirubin on the animals’ body weight

**Fig. 3:**
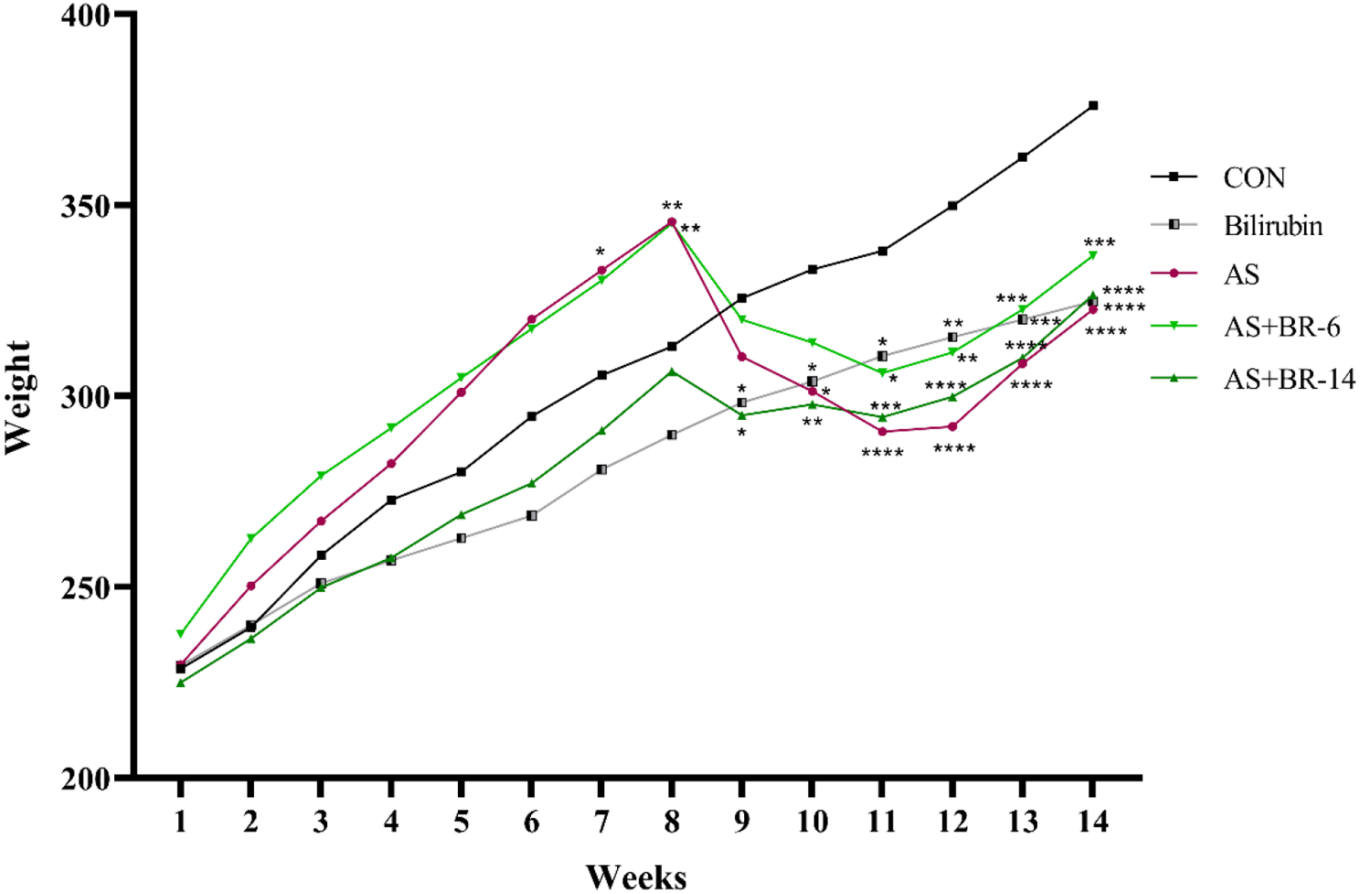
Body weight changes of the rats during 14 weeks of treatment. Based on two-way repeated measures ANOVA analysis, there was a significant interaction between the effect of treatment and the time on body weight. Values are expressed as mean ± SD (*n* = 6). Control = Normal rats; Bilirubin = Healthy rats received bilirubin; AS = Atherosclerosis model; AS + BR-6 (a.k.a. Treated group) = Atherosclerosis model received HFD + STZ + bilirubin (for 6 weeks); AS + BR-14 = Atherosclerosis model received HFD + STZ + bilirubin (for 14 weeks) (a.k.a. Protected group). * The control vs. other groups. *, **, *** and **** indicate *p* < 0.05, *p* < 0.01, *p* < 0.001, and *p* < 0.0001, respectively

The body weight changes of the experimental rats over 14 weeks are shown in Fig. 3. According to the two-way repeated ANOVA data, the body weight of the animal during the 14 weeks experiment reported a significant increase (*p* < 0.0001), except the control group. The average body weight of the AS and AS + BR-6 rats indicated a significant increase after eight weeks of dietary manipulation compared to the control group (*p* < 0.05). Also, the weight gain procedure in the B group was slower than in the control group, and this difference was significant beginning the ninth week (*p* < 0.05). Although weight quantities were declined following the STZ injection in the AS and AS + BR-6 groups compared to the eighth week (*p* < 0.0001 and *p* < 0.01, respectively), no significant weight loss was observed in the AS + BR-14 group, which received bilirubin from the beginning.

### Effects of bilirubin therapy on FBG and lipid content

**Fig. 4:**
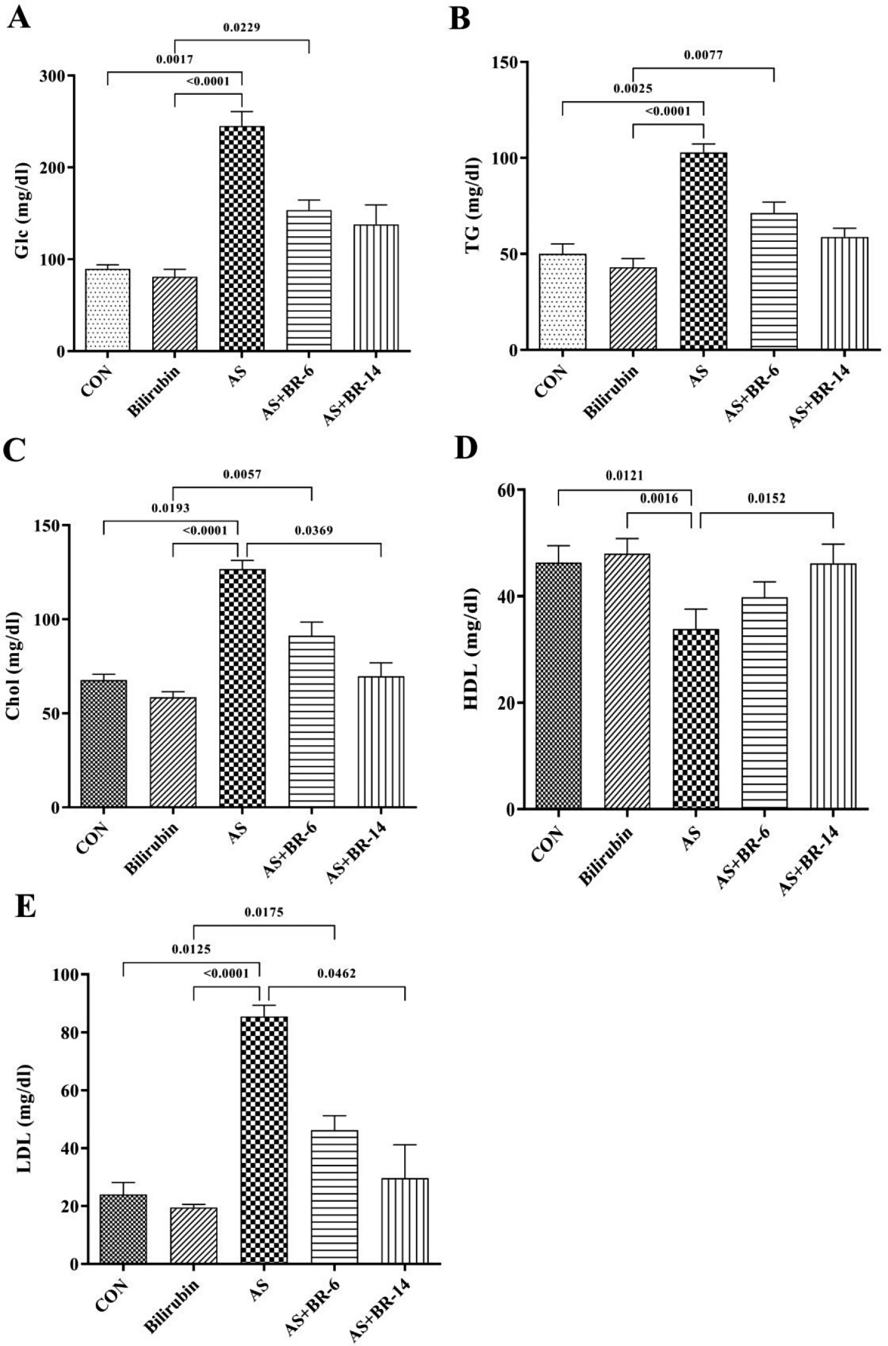
Serum glucose and lipid content among different group. **(A)** Glucose, **(B)** TG, **(C)** Chol, **(D)** HDL-Chol, **(E)** LDL-Chol. Control = Normal rats; Bilirubin = Healthy rats received bilirubin; AS = Atherosclerosis model; AS + BR-6 (a.k.a. Treated group) = Atherosclerosis model received HFD + STZ + bilirubin (for 6 weeks); AS + BR-14 = Atherosclerosis model received HFD + STZ + bilirubin (for 14 weeks) (a.k.a. Protected group). All values ​​are expressed as mean ± standard deviation (*n* = 6). Statistical analysis was determined using the Kruskal-Wallis test with Dunn’s multiple comparisons post hoc analysis

Serum levels of glucose, TG, Chol, and LDL-Chol were increased in AS rats compared to the control group, whereas HDL-Chol level was decreased (*p* < 0.0001). Bilirubin administration induced a significant decrease in serum glucose and lipid parameters in AS + BR-6 and AS + BR-14 groups (*p* < 0.0001), except for HDL-Chol. (Fig. 4A, B, C, D,and E).

### Bilirubin improved the liver markers

**Fig. 5:**
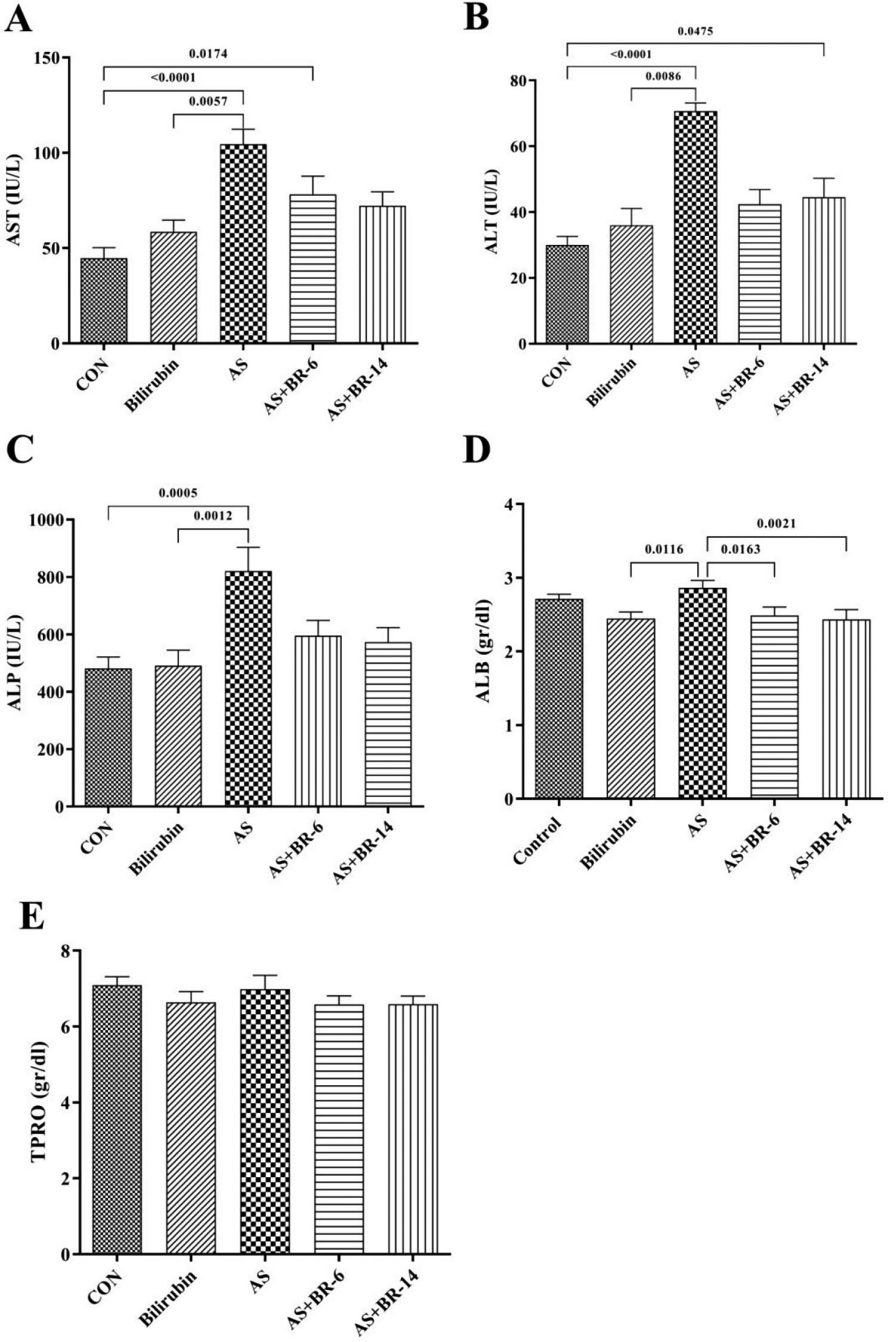
Serum levels of liver function indices among different groups. **(A)** AST, **(B)** ALT, **(C)** ALP, **(D)** Alb, **(E)** TP. Control = Normal rats; Bilirubin = Healthy rats received bilirubin; AS = Atherosclerosis model; AS + BR-6 (a.k.a. Treated group) = Atherosclerosis model received HFD + STZ + bilirubin (for 6 weeks); AS + BR-14 = Atherosclerosis model received HFD + STZ + bilirubin (for 14 weeks) (a.k.a. Protected group). All values ​​are expressed as mean ± standard deviation (*n* = 6). Statistical analysis was determined using the Kruskal-Wallis test with Dunn’s multiple comparisons post hoc analysis

The liver markers indicated a significant increase in levels of AST, ALT, ALP, and Alb compared to the control group (*p* < 0.05). Bilirubin administration reported a significant reverse in the levels of aforementioned parameters in the AS + BR-6 and AS + BR-14 groups compared to the AS rats (*p* < 0.05) (Fig. 5A, B, C, **and D**). The TP levels in AS group, did not represent any significant changes compared to the other groups (Fig. 5E).

### The status of serum bilirubin profile (TBIL, DBIL, and IBIL) after bilirubin administration

**Fig. 6:**
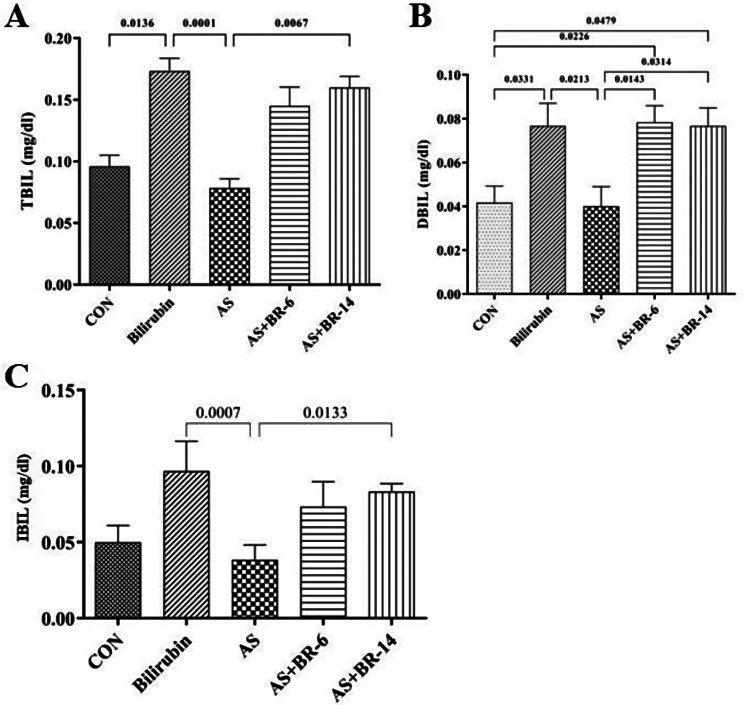
Serum levels of bilirubin profile among different groups. **(A)** TBIL, **(B)** DBIL, **(C)** IBIL. Control = Normal rats; Bilirubin = Healthy rats received bilirubin; AS = Atherosclerosis model; AS + BR-6 (a.k.a. Treated group) = Atherosclerosis model received HFD + STZ + bilirubin (for 6 weeks); AS + BR-14 = Atherosclerosis model received HFD + STZ + bilirubin (for 14 weeks) (a.k.a. Protected group). All values ​​are expressed as mean ± standard deviation (*n* = 6). Statistical analysis was determined using the Kruskal-Wallis test with Dunn’s multiple comparisons post hoc analysis

Serum levels of TBIL and DBIL in the AS + BR-6 and AS + BR-14 groups showed a remarkable elevation compared to the control and AS groups (*p* < 0.05) (Fig. 6A, B). All bilirubin-receiving groups showed a significant increase in the calculated levels of indirect bilirubin (IBIL) compared to the control and AS groups (Fig. 6C).

### Bilirubin administration protectively prevented the adhesion molecule expression and attenuated the LOX-1 and iNOS expression

**Fig. 7:**
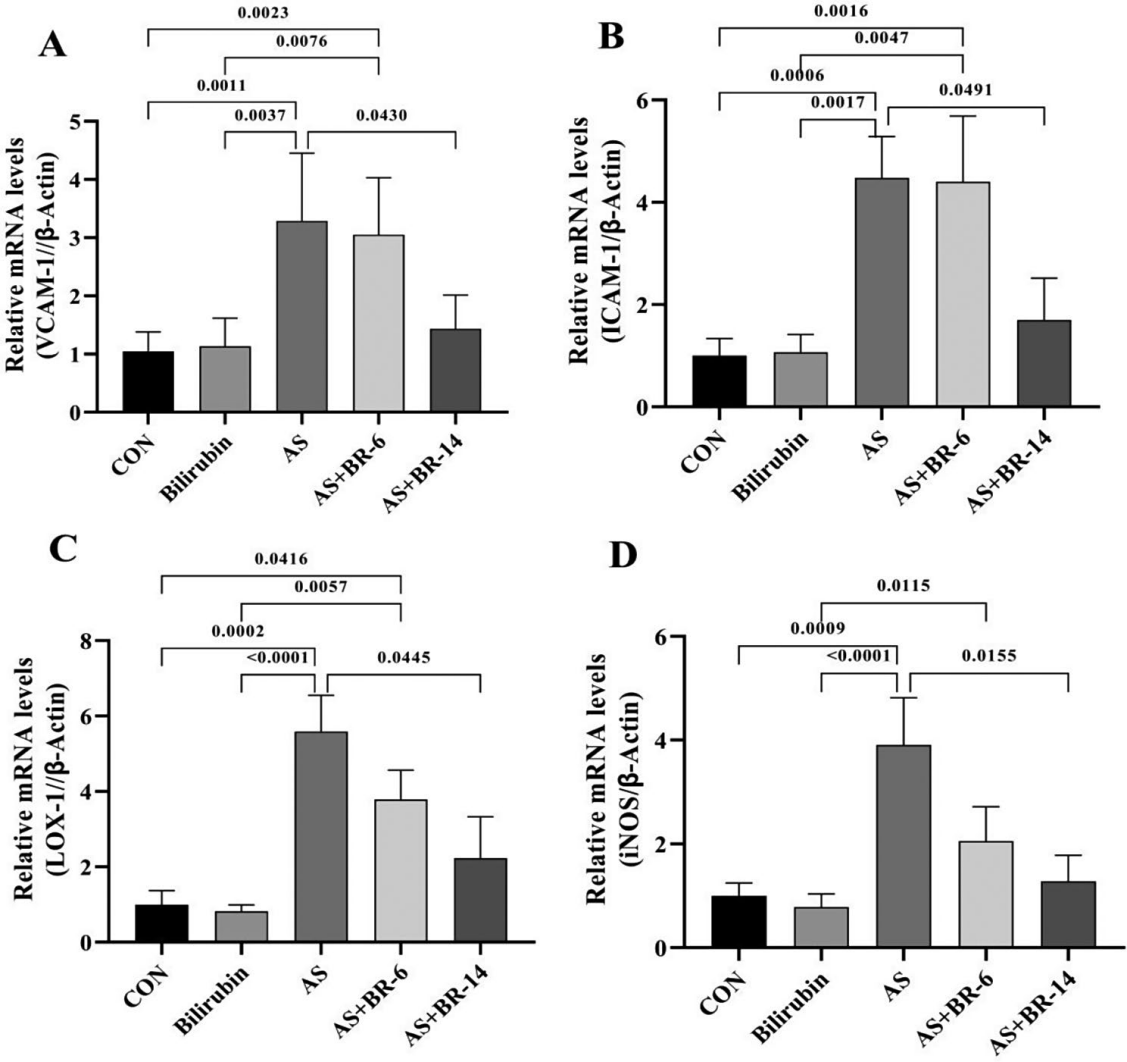
Relative mRNA expression levels of VCAM-1, ICAM-1, LOX-1, and iNOS genes in the aorta tissue excised from the rats. **(A)** VCAM-1, **(B)** ICAM-1, **(C)** LOX-1, and **(D)** iNOS. Control = Normal rats; Bilirubin = Healthy rats received bilirubin; AS = Atherosclerosis model; AS + BR-6 (a.k.a. Treated group) = Atherosclerosis model received HFD + STZ + bilirubin (for 6 weeks); AS + BR-14 = Atherosclerosis model received HFD + STZ + bilirubin (for 14 weeks) (a.k.a. Protected group). All values ​​are expressed as mean ± standard deviation (*n* = 6). Statistical analysis was determined using the Kruskal-Wallis test with Dunn’s multiple comparisons post hoc analysis

The expression levels of VCAM-1 and ICAM-1 were remarkably increased in the AS group compared to the control rats (*p* < 0.05). Although bilirubin administration did not significantly change the expression pattern of VCAM-1 and ICAM-1 in the AS + BR-6 groups compared to the AS rats, it significantly prevented the expression of the corresponding genes in the AS + BR-14 group, indicating the protective potential of bilirubin (*p* < 0.05). LOX-1 and iNOS expression levels were also significantly increased in the AS group compared to the control rats (*p* < 0.05). Bilirubin administration effectively decreased the expression of LOX-1 and iNOS genes in the AS + BR-14 group in comparison to the AS rats (Fig. 7A, B, C, **and D**).

### Weight and volume of the left ventricle following the bilirubin administration

**Fig. 8:**
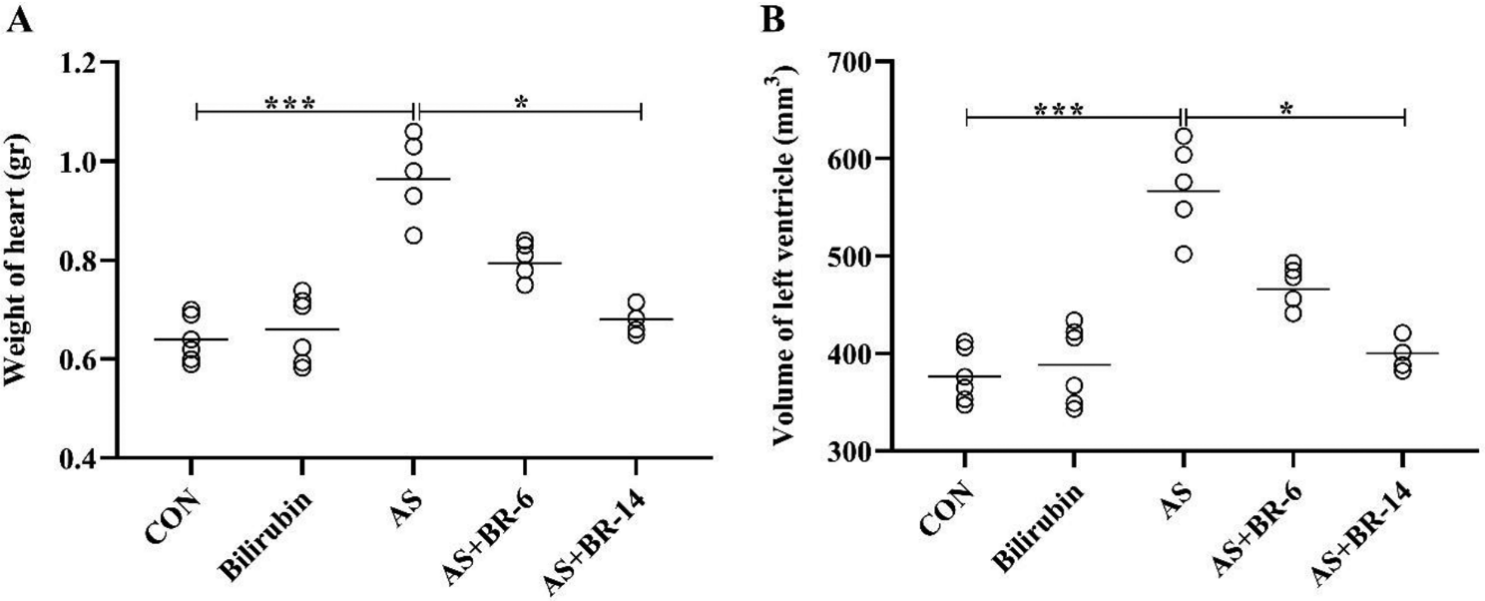
The effects of bilirubin on the heart weight and the left ventricle’s volume. Control = Normal rats; Bilirubin = Healthy rats received bilirubin; AS = Atherosclerosis model; AS + BR-6 (a.k.a. Treated group) = Atherosclerosis model received HFD + STZ + bilirubin (for 6 weeks); AS + BR-14 = Atherosclerosis model received HFD + STZ + bilirubin (for 14 weeks) (a.k.a. Protected group). All values ​​are expressed as mean ± standard deviation (*n* = 6). Statistical analysis was determined using the Kruskal-Wallis test with Dunn’s multiple comparisons post hoc analysis

The effects of bilirubin on heart weight and the left ventricle’s volume are shown in Fig. 8. The mean weight of the heart and volume of the left ventricle reported a significant increase in the AS group compared to the control group (*p* < 0.001) (Fig. 8A, B). Comparing to the AS group, the left ventricular volume and heart weight showed a decrease in both AS + BR-6 and AS + BR-14 groups (*p* < 0.05).

### The total volume of the heart structures

**Fig. 9:**
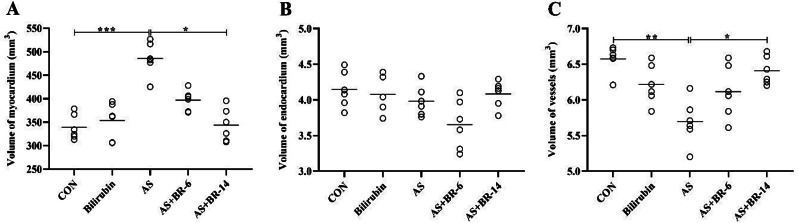
The effects of bilirubin on the total volume of heart structures. **(A)** Myocardium **(B)** Endocardium, **(C)** Vessels. Control = Normal rats; Bilirubin = Healthy rats received bilirubin; AS = Atherosclerosis model; AS + BR-6 (a.k.a. Treated group) = Atherosclerosis model received HFD + STZ + bilirubin (for 6 weeks); AS + BR-14 = Atherosclerosis model received HFD + STZ + bilirubin (for 14 weeks) (a.k.a. Protected group). All values ​​are expressed as mean ± standard deviation (*n* = 6). Statistical analysis was determined using the Kruskal-Wallis test with Dunn’s multiple comparisons post hoc analysis

The heart-related quantitative parameters of the left ventricle structures (myocardium, endocardium, and the vessels) are illustrated in Fig. 9. The AS rats represented myocardial hypertrophy, an increase of 43.10%, compared to the control group. These parameters showed a remarkable decline in the AS + BR-14 rats in comparison to the AS group (*p* < 0.05). Although there was a decrease in the AS + BR-6 group compared to the AS group, it was not statistically significant (Fig. 9A). There was no difference between the control and the AS rats, regarding the total volume of the endocardium (Fig. 9B). Considering the myocardial vascularization, the results of the evaluation of total volume of the vessels, as a substantial parameter to determine myocardial vitality, indicated that the intramyocardial vessels’ volume was significantly decreased in the AS group versus the control group (*p* < 0.05) (Fig. 9C).

### Histopathological findings

**Fig. 10:**
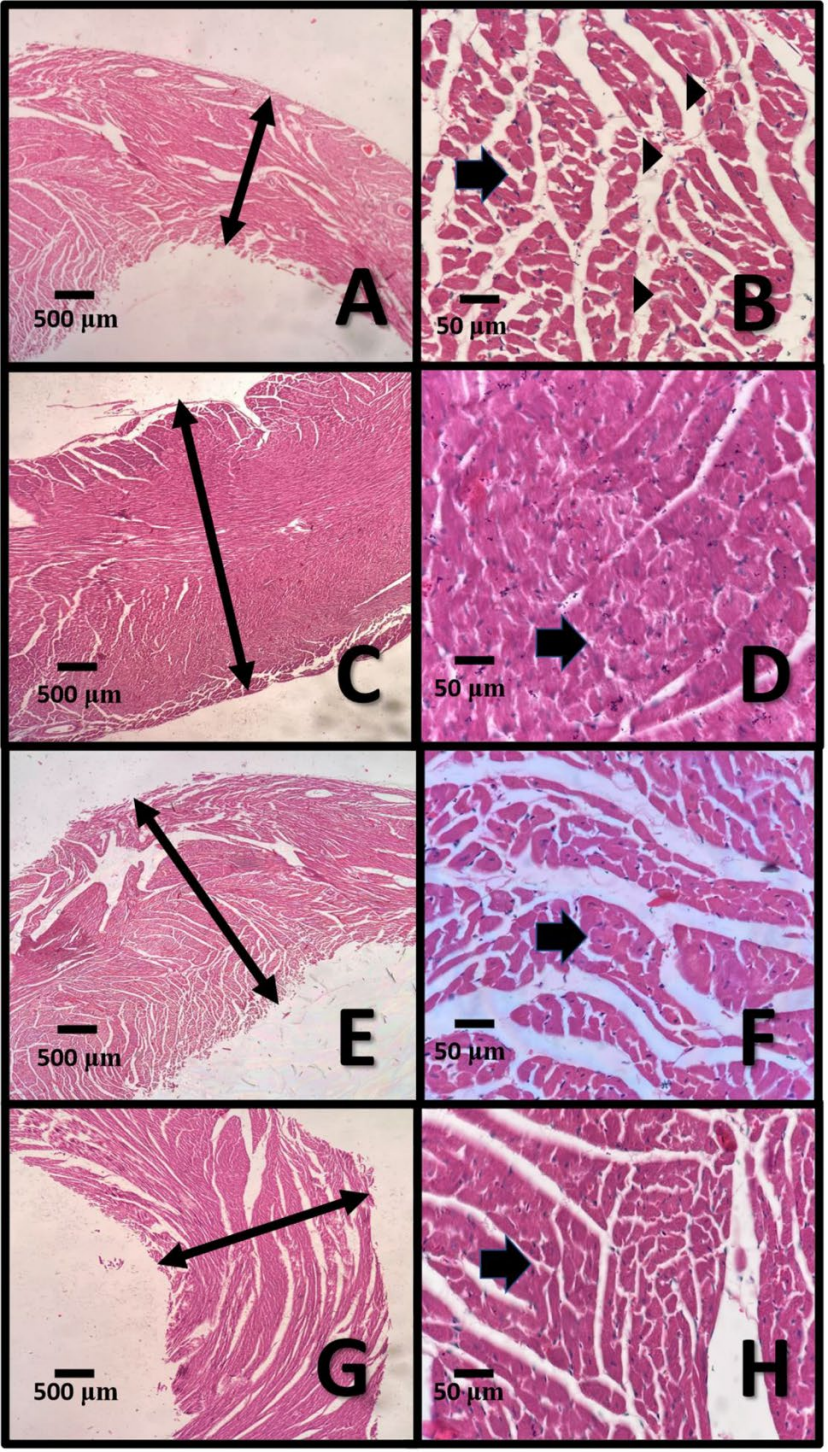
Histopathology sections of the left ventricles collected from different groups of rats.(** A, B**) Histopathology sections of the left ventricle of the heart of control group shows myocardial layer (double headed arrow) with bundles of cardiomyocytes (arrow) with reasonable loose vascular connective tissue (arrowheads) between cardiac muscle bundles (X40 (left side) and X400 (right side); Hematoxyllin and Eosin stain); (**C, D**) Histopathology sections of the left ventricle of the heart of group B shows hypertrophied thickened myocardial layer (double headed arrow) with enlargement and hypertrophy of bundles of cardiomyocytes (arrow) with remarkable loss of connective tissue between cardiac muscle bundles and decrease in vascularity (X40 (left side) and X400 (right side); Hematoxyllin and Eosin stain); (**E, F**) Histopathology sections of the left ventricle of the heart of group C shows relatively normal myocardial layer (double headed arrow) with bundles of cardiomyocytes (arrow) with loose vascular connective tissue between cardiac muscle bundles (X40 (left side) and X400 (right side); Hematoxyllin and Eosin stain); (**G, H**) Histopathology sections of the left ventricle of the heart of group D shows relatively normal myocardial layer (double headed arrow) with bundles of cardiomyocytes (arrow) with loose highly vascular connective tissue between cardiac muscle bundles (X40 (left side) and X400 (right side); Hematoxyllin and Eosin stain)

Histopathological evaluations of the left ventricle’s tissue revealed a significant increase (*p* < 0.05) in the thickness of the myocardial layer, as well as the hypertrophy of cardiomyocytes’ bundles, along with the remarkable loss of connective tissue between cardiac muscle bundles and a decrease in vascularity in the AS group compared to the control group (Fig. 10A-D). Whilst, bilirubin therapy showed a reduction in the thickness of the myocardial layer and the bundles of cardiomyocytes with loose vascular connective tissue between cardiac muscle bundles in the left ventricle of the heart of the AS + BR-6 and AS + BR-14 groups compared to the AS (*p* < 0.05) (Fig. 10E- H).

## Discussion

Several studies have recently focused on the beneficial effects of bilirubin. The high failure rate in drug development necessitates efficient pre-clinical evaluation. Bilirubin, a ubiquitous molecule in eukaryotes, offers a unique opportunity. Its natural presence in humans allows leveraging existing data from epidemiological studies with elevated bilirubin levels (Gilbert’s Syndrome) to assess potential health effects. This condition, affecting 5–10% of the population with mildly elevated bilirubin (1–6 mg/dl), provides valuable insights without requiring additional clinical trials. Mutations in the uridine diphosphate glucuronosyltransferase 1 A (UGT-1 A) gene, responsible for bilirubin conjugation, cause this syndrome. Animal models like Gunn rats further support these investigations. Decades of research on individuals with Gilbert’s Syndrome reveal a lower risk of coronary artery disease, stroke, and improved outcomes after organ transplantation. These findings suggest potential therapeutic benefits of elevated bilirubin without adverse effects. Criggler-Najar Syndrome, as a rare defect, also highlights the importance of dose response. High bilirubin levels (> 20 mg/dL) observed in this case lead to severe neurotoxicity. This approach provides valuable safety, dose-response, and efficacy data that would conventionally require extensive clinical trials. The natural presence of bilirubin in humans combined with available epidemiological data streamlines drug development for potential therapeutic applications [36, 37].

In this area of expertise, we previously demonstrated that bilirubin could ameliorate the pathological conditions related to common metabolic diseases and cancers [38–41]. Herein, our findings clearly showed that bilirubin might also exert protective and therapeutic effects on atherosclerosis. Consistently, individuals with mild hyperbilirubinemia, especially those with Gilbert’s syndrome, have been found to experience a lower risk of certain CVDs, such as CAD and ischemic heart diseases [42].

We noticed that bilirubin declined the serum levels of Chol, TG, and oxidative lipid-to-protein ratio as major risk factors for atherosclerosis and CVD in rat model of atherosclerosis. The ligation of VCAM-1 and ICAM-1 with their corresponding integrin leads to the activation of NADPH oxidase (NOX) and xanthine oxidase (XO). These enzymes produce the ROS, including the superoxide anion (O_2_^• −^) and hydrogen peroxide (H_2_O_2_) [43, 44]. The subsequent signaling cascade activates matrix metalloproteinases (MMP)-2 and − 9, resulting in the disruption of endothelial tight junctions. A further effect of oxidative stress is characterized by the direct action of free radicals’ overproduction on eNOS, which causes eNOS uncoupling, and thus the impaired NO production, which gives rise to the increased superoxide anions generation and oxidative damage. Hence, the decreased NO levels beside the increased lipoperoxide formation can up-regulate the iNOS expression to compensate the attenuated NO bioavailability [45]. Notwithstanding, lipoperoxides could exert a detrimental effect on endothelial cells in response to hypercholesterolemia. Indeed, an increase in the levels of lipoperoxides leads to an augmented expression of LOX-1, and consequently further defect in NO production through the NOX activation, as a vicious circle [19, 46, 47]. Based on previous investigations, local NO release is lower in patients with serum levels of LDL-Chol up to 160 mg/dl [48]. The interruption of the above-stated vicious circle might explain the protective and therapeutic effects of bilirubin on atherosclerosis suppression.

Although bilirubin administration did not alter the mRNA expression levels of adhesion molecules in the aorta, it prevented from their overexpression during receiving HFD, suggesting the protective effects of bilirubin against atherogenesis progression. In addition, bilirubin scavenges NOX- and XO-derived ROS, through activating the biliverdin reductase (BVR) that reduces the ox-LDL production [7, 49]. Our findings indicated that LOX-1 overexpression in atherosclerotic rats could desirably be reversed by bilirubin administration. Mollace et al. (2015) indicated that silencing the LOX-1 receptor by short hairpin RNA (shRNA) provided a type of protection against ox-LDL-induced apoptosis and restored the autophagy [50]. These findings confirm the crucial role of LOX-1 in mediating the ox-LDL-dependent impairment of autophagy and suggest another possible pathway targeted by bilirubin. Parallel outcomes have also been achieved in animal models of atherosclerotic disorders in carotid arteries. It has been declared that LOX-1-induced ox-LDL uptake induces the endothelial dysfunction observed in the early stages of this pathological condition [51, 52]. In our experiment, we observed that bilirubin could remarkably suppress the LOX-1 overexpression and decrease serum LDL levels in atherosclerotic rats. Accordingly, bilirubin can prevent atherosclerosis progression via interrupting the ROS and ox-LDL production, and their further interaction with LOX-1.

Regarding the microbial assessments performed on *Escherichia coli* (E. coli) lipopolysaccharide (LPS), LPS could promote the LOX-1 mRNA expression through the TLR4/MyD88/ROS-activated p38MAPK/NF-κB pathway in endothelial cells [53], and based on our finding, this pathway might be a new possible regulatory mechanism for bilirubin through inhibiting the LOX-1 expression and atherosclerosis development. The enhanced activation and overexpression of iNOS has also been observed through the TLR4-dependent pathway in LPS-induced human umbilical vein endothelial cells (HUVECs), due to LOX-1 overexpression [46]. Peroxynitrite (PNT), which is an undesirable product generated at high levels during iNOS activity, has been reported to be associated with endothelial cells death, i.e. apoptosis, as demonstrated by the enhancement of the caspase-3 expression and there are also correlations between HO-1 and the reduced caspase-3 [54, 55]. The q RT-PCR analyses revealed a noticeable decrease in iNOS mRNA expression of rats that received bilirubin compared to the AS group, and thus the protective effects of bilirubin might be the consequence of the inhibition of PNT production by decreasing iNOS expression and subsequent decrease in caspase-3 expression [46, 56]. .

Beyond the biochemical and gene expression analyses, our stereological assessments of the animals’ heart demonstrated an increase in heart weight in HFD-fed groups, indicating cardiomegaly. The overfed animals were also found with left ventricular hypertrophy with an increased volume of myocardium and a decreased vessel volume. These findings were approved by histopathological observations as well. We noticed that bilirubin treatment led to a decrease in the thickness of the myocardial layer in the left ventricle of the heart. Stereologically, the left ventricular volume and heart weight were normal following the bilirubin administration. Together, these cardioprotective and preventive effects of bilirubin against atherosclerosis may be attributed to anti-inflammatory properties of bilirubin, along with its antioxidant and anti-apoptotic characteristics that have been observed in previous studies. In line with our outcomes, Moreira and colleagues declared consistent findings on the effect of over-nutrition on the heart morphology and stereology of obese animals. They showed that heart weight/tibia length ratio was increased and left ventricular hypertrophy occurred with an increased area of cardiomyocytes and a decreased vessel density in the heart of over-fed pups [57]. A cohort study on patients with type 2 diabetes mellitus conducted by Tomoaki Inoue et al., showed that the serum bilirubin concentration might be associated with the progression of concentric left ventricular remodeling in patients with type 2 diabetes mellitus. They reported that the patients with lower bilirubin levels had a higher prevalence of concentric left ventricular remodeling compared with those with higher bilirubin levels [58]. Literally, bilirubin is supposed to mitigate structural and histopathological damage in the heart and vasculature through several mechanisms. It effectively neutralizes harmful ROS, thereby reducing oxidative stress that can damage cardiac and vascular cells. This antioxidant function preserves cellular integrity and prevents oxidative modifications to lipids, proteins, and DNA within these tissues. Additionally, bilirubin’s ability to suppress pro-inflammatory molecules and lessen inflammatory cell infiltration decreases inflammation-induced damage, leading to a reduction in associated histopathological changes. Furthermore, bilirubin enhances endothelial function, a critical factor for vascular health, by promoting nitric oxide availability and reducing endothelial dysfunction. It also modulates the immune response by downregulating adhesion molecules and chemokines, which are involved in immune cell recruitment, thereby protecting against vascular inflammation and atherosclerosis. Beyond these effects, bilirubin influences cell signaling pathways governing cell growth, apoptosis, and fibrosis, contributing to the maintenance of cardiac and vascular structure and function. Notably, bilirubin can regulate genes associated with stress responses, metabolism, and inflammation, potentially adding to its protective effects. Collectively, these mechanisms position bilirubin as a potential therapeutic agent for cardiovascular diseases by protecting against and improving structural and histopathological changes in the heart and vasculature [59–62].

To achieve a more comprehensive assessment of the effects of bilirubin on preventing diabetic and obesity-related cardiovascular diseases, measuring the levels and expression of inflammatory markers can provide a more accurate insight into the bilirubin’s functionality. Moreover, focusing on the negative effects of streptozotocin and the role of bilirubin in preventing its adverse effects can be a subject for future studies.

## Conclusion

The administration of bilirubin was found to have remarkable efficacy in mitigating structural changes in the hearts of atherosclerotic rats. Bilirubin also demonstrated therapeutic and protective effects on atherosclerosis-related genetic and biochemical parameters, particularly during HFD feeding. Furthermore, bilirubin treatment led to a decrease in the thickness of the myocardial layer in the left ventricle of the heart. Stereologically, the left ventricular volume and heart weight were normal following the bilirubin administration. Together, these cardioprotective and preventive effects of bilirubin against atherosclerosis may be attributed to anti-inflammatory properties of bilirubin, along with its antioxidant and anti-apoptotic characteristics that have been observed in previous studies. Findings of our study highlight the promising role of bilirubin as an endogenous protective/curative compound, warranting further investigations in this field.

## Data Availability

All datasets analyzed during the current study are available from the corresponding author on reasonable requests.
